# Pharmaceutical care program for patients with chronic kidney disease in the community pharmacy: Detection of nephrotoxic drugs and dose adjustment. Viability study

**DOI:** 10.1371/journal.pone.0278648

**Published:** 2022-12-22

**Authors:** Gema Escribá-Martí, Iker Cámara-Ramos, María Teresa Climent-Catalá, Verónica Escudero-Quesada, Luis Salar-Ibáñez

**Affiliations:** 1 Community Pharmacist in Segart, Valencia, Spain; 2 Community Pharmacist in Bilbao, Bilbao, Spain; 3 Doctor of Pharmacy, President of the SEFAC Delegation in the Valencian Community, Community Pharmacist in L’Olleria, Valencia, Spain; 4 Nephrologist, Nephrology Service, Dr. Peset University Hospital, Valencia, Spain; 5 Doctor of Pharmacy, Cardenal Herrera University–CEU, Community Pharmacist in Valencia, Valencia, Spain; Istituto Di Ricerche Farmacologiche Mario Negri, ITALY

## Abstract

**Introduction:**

Chronic kidney disease (CKD) is a major health problem. Early detection is the key to reducing morbidity and mortality, but it is difficult because it occurs without symptoms. Diagnosis of CKD is also important to avoid nephrotoxic drugs and to adjust the doses of other medications that may be affected. Pharmacies, due to their proximity to the population, frequency of patient visits, and knowledge of medication use are an ideal location for point-of-care diagnosis or CKD.

**Objective:**

To detect and refer to the primary care physician patients with low estimated glomerular filtration rate (eGFR) who use nephrotoxic drugs or who may require a dose adjustment.

**Methodology:**

Pharmacy users over 60 years of age who agreed to participate were given a creatinine/eGFR test with a point-of-care meter. The eGFR was calculated and if it was less than 60 ml/min/1.73 m2, their medications were evaluated to identify nephrotoxic drugs or drugs that potentially required adjustment. If either were found, they were referred to their doctor for further management.

**Results:**

198 patients were recruited in 4 pharmacies, of which 87 (43.9%) had an eGFR less than 60 ml/min/1.73 m2. They were taking a total of 635 medications. Of these 635 medications, 50 (7.9%) were affected by kidney function. Dose adjustment was recommended in 31 and discontinuation in 19. The primary care doctor accepted the recommendations for 14 medications: dose adjustment for 6 and withdrawal in 8. This represents 2.3% of medications taken by patients with an eGFR less than 60 ml/min/1.73 m2. The 50 medications identified were taken by 29 patients (33.3% of the 87 with a low eGFR) and a change in treatment was generated in 9 patients, representing 4.6% of the total number of patients in the sample, and 10% of the patients with a low eGFR.

**Conclusion:**

Point-of-care testing for kidney function in a pharmacy setting is feasible and identifies a significant number of patients with eGFR under 60 ml/min/1.73 m^2^. It also allows for appropriate medication management recommendations in this patient group.

## Introduction

Chronic Kidney Disease (CKD) in adults is defined as the “*presence of a kidney structural or functional alteration (sediment*, *image*, *histology) that persists for more than 3 months*, *with or without deterioration of kidney function; or a glomerular filtration rate (GFR) < 60 ml/min/1*.*73 m* ^*2*^ *without other signs of kidney disease”* [1].

CKD is a major global health problem. In a World Health Organization (WHO) publication it is considered "*the most neglected chronic disease*" [2] with a worldwide prevalence estimated at 13.4% (95% CI 11.7–15.1). The most frequent stage is 3a (GFR between 45 and 60 ml/minute/1.73 m ^2^) with 7.6% of prevalence (6.4–8.9) [3]. In Spain, with data obtained from the ENRICA (Estudio de Nutrición y Riesgo Cardiovascular en España) study, 15.1% (14.3–16.0) of the population has CKD, with stage 3a also being the most prevalent (10.0% CI95% 9.3–10, 8). It is three times higher in men than in women and increases markedly with age, reaching 37.3% in those over 65 years of age [4, 5]. Over time, the kidneys progressively lose their filtering capacity. It is estimated that the mean annual decrease in GFR is 0.8 ml/min/1.73m^2^ in women and 1.4 ml/min/1.73m^2^ in men [6]. Other than age, diabetes and hypertension are the most important risk factors for developing CKD [7]. Other risks include obesity, dyslipidemia, smoking, hyperuricemia, hypoalbuminemia, and cardiovascular disease, [1, 8] many of which are also common in the elderly population.

The most frequent complications of CKD are cardiovascular disease, anemia and osteoporosis [9]. Approximately 1% of patients with CKD will eventually require *kidney* replacement therapy, either dialysis or kidney transplant, but this represents a significant reduction in the quality and life expectancy of these patients [4]. According to the National Transplant Organization in Spain, 2423 kidney transplants were performed in 2019 [10].

Early detection of the disease is the key to preventing morbidity and mortality, but this is difficult because in the early stages it is usually asymptomatic. It is in primary care that these asymptomatic patients can be detected [11], especially patients with hypertension, diabetes, cardiovascular disease or a family history of CKD.

Treatment consists of protecting the kidney by controlling the complications that may arise and the risk factors that can aggravate the disease, especially hypertension and diabetes [12]. In addition, identifying and managing nephrotoxic drugs in stage 3 CKD, (Table 1) and discontinuing them in stages 4–5 [11–13] is important.

**Table 1:** Classification of CKD by categories according to glomerular filtration rate.

Stage	ml/min/1.73m2)
G1	≥ 90
G2	60–89
G3a	45–59
G3b	30–44
G4	15–29
G5	<15

This includes adjusting the dose of drugs based on eGFR to avoid further nephrotoxicity, or potential overdosing due to decreased *kidney* clearance [13–15]. Studies have shown that up to 52% of patients who need some adjustments are detected [14].

Due to its accessibility and availability, the community pharmacy could be considered an extension of the primary care service. It is increasing its healthcare capabilities in Spain and in other countries, especially in Canada and the Netherlands. Among them are services related to CKD, including screening and detection of asymptomatic patients [16], advice and reconciliation of medication in patients at hospital discharge [17], subsequent follow-up to detect medication related complications, including non-compliance [18]. In addition, a 2019 study used POCT measurement of *kidney* function (GFR) in community pharmacies in the Netherlands to prevent hospitalizations related to antibiotic adverse events [19]. In this study, the authors showed an annual saving of €86 per patient thanks to the possibility of measuring *kidney* function in Dutch community pharmacies. The savings were mainly due to hospitalizations avoided.

The community pharmacy is very well placed to carry out screening, as has already been repeatedly demonstrated in Spain and in other countries [20–22]. Two million people pass through Spanish pharmacies every day [23] and many of these people do not typically go to the doctor, which makes it an ideal place to detect occult diseases, including CKD.

Specifically for CKD, the community pharmacy can benefit the Public Health System and the population through the screening and early detection of CKD and the review of their medication to detect the inappropriate use of nephrotoxic drugs and propose dose adjustment for medications that are cleared by the kidneys [24].

Is Spain, the problem of insufficient communication between the Spanish community pharmacy and the Public Health System is known and widely recognized. Unfortunately, there is no direct communication between community pharmacy and primary care medicine. Therefore, the only information available for the participating pharmacists is what is prescribed in the electronic prescription system and what the patient can and wants to tell.

### Goals

- Detect and refer to the doctor patients with low GFR (<60 ml/min/1.73 m ^2^).
- Detect and notify the doctor of the use of nephrotoxic drugs in these patients.
- Detect and notify the doctor of the use of excessive doses according to the GFR of these patients.
- To study the capacity of the Spanish community pharmacy to collaborate in the adaptation of treatments in patients with possible kidney disease.

## Material and methods

This is non-controlled prospective, follow-up, experimental, analytical multicenter study. Four community pharmacies participated, three from the Valencian Community and one from the Basque Country. Each was required to recruit a minimum of 50 patients.

### Patient inclusion criteria

Patients over 60 years of age with a BMI between 19 and 35 kg /m^2^, who visited the pharmacies for any reason and either, use at least one nephrotoxic medication, or one that may require dose adjustment based on eGFR. The patient had to agree to participate in the study signing the written informed consent.

### Exclusion criteria

■ Patients with special diets (strict vegetarians, creatinine or creatine supplementation) or malnutrition
■ Changes in muscle mass (amputations, loss of muscle mass, muscle diseases or paralysis)
■ Severe liver disease, generalized edema, or ascites
■ Acute *kidney* failure
■ Patient on dialysis or waiting for a kidney transplant.

Patients who met the criteria were asked if they had an eGFR measurement in the previous 3 months. If they did not have it, they were offered a point-of-care test for creatinine in capillary blood with the StatSensor Xpress® (Nova Biomedical, Waltham, MA) analyzer and the eGFR was calculated using the CKD-EPI formula. This analyzer was previously validated for this study [25].

If the GFR was less than 60 ml/min/1.73 m^2^, the patient’s medication was evaluated for nephrotoxic drugs or drugs that need dose adjustment using the ChekTheMeds ® web application [26], Consensus Guide for the use of drugs in kidney failure [27] and the technical data sheets of the drugs involved.

If any of these medications were found, a report was generated for the Primary Care Physician (MAP), which was given to the patient in an open envelope.

Subsequently, information was collected on any changes made by the PCP as a result of our intervention.

The study was approved by the CEIM (Medical Research Ethics Committee) of the Dr. Peset University Hospital under number CEIm:66/19

At the end of the study, pharmacies estimated the time spent on each patient. Patients were divided into 3 categories:

- Those who did not require a medication study
- Those who required a medication study
- Those referred to a doctor.

### Statistical analysis

Before analysis, the data was anonymized to maintain the confidentiality of the patients.

Descriptive statistics using mean and median are used. For comparisons, the χ ^2^ was used for qualitative variables and Student’s t-test with equal or different variances for quantitative variables. Differences are considered significant when p<0.05

## Results

Four community pharmacies participated between April 11 and July 24, 2019. The goal was to recruit 200 patients, 50 per pharmacy. Participation in the study was offered to 207 patients of whom 7 (3.4%) did not accept it and 2 had to be discarded due to errors in the registry. Therefore, the sample is 198 patients. One hundred (50.5%) were women. The mean age was 72.6 years (SD 8.0), there was no difference in age based on sex (p = 0.139).

Of the 198 patients, 16 had a creatinine value in the previous 3 months; the remaining (91.9%) had their creatinine and eGFR by capillary blood analysis using the StatSensor Xpress ® analyzer.

The 198 patients were taking a total of 1234 medications (mean 6.2 SD 3.4 range 1–19). There was no difference in the number of medications based on gender (p = 0.364)

87 patients (43.9%) had an eGFR < 60 ml/min/1.73 m^2^. There was no difference according to gender (p = 0.259).

Table 2 shows the distribution of patients by age group together with the eGFR of the group (mean; SD) and the distribution of patients in each stage of CKD.

**Table 2:** Distribution of patients by age group and proportion of patients according to their chronic kidney disease stage.

Age	No. (%)	Average GFR ml/min/1.73 m ^2^ (SD)	CKD stage			
			1–2 (GFR >60 ml/min/1.73 m ^2^)	3a (FG 45–60 ml/min/1.73 m ^2^)	3b (FG 30–45 ml/min/1.73 m ^2^)	4–5 (GFR <30 ml/min/1.73 m ^2^)
			No. (%)	No. (%)	No. (%)	No. (%)
60–70	85 (42.9)	78.4 (33.9)	57 (67.1)	22 (25.9)	5 (5.9)	1 (1.2)
71–80	78 (39.4)	67.2 (31.9)	40 (51.3)	22 (28.2)	12 (15.4)	4 (5.1)
>81	35 (17.7)	57.4 (17.1)	14 (40.0)	13 (37.1)	8 (22.9)	0 (0.0)
Total	198 (100)	70.3 (31.6)	111 (56.1)	57 (28.8)	25 12.6)	5 (2.5)

(SD Standard Deviation, GFR Glomerular Filtration in mL/min, CKD Chronic Kidney Disease).

Patients with GFR > = 60 ml/min/1.73 m^2^ were younger (70.9 years SD 7.7) and took fewer medications (5.4 medications SD 2.9) than those with eGFR <60 ml/min/1.73 m^2^ (74.9 years SD 7.9; 7.3 drugs SD 3.7) (p _age_ = 0.0004; p _drugs_ = 0.0001). The detail broken down by CKD stage can be seen in Table 3.

**Table 3:** Patients and medications grouped according to CKD stage.

CKD/ GF stage	Patients		Medicines	
	n	Age (SD)	n	Mean per patient (SD)
4-5/<30	5	74.0 (4.4)	46	9.2 (3.1)
3b/30-45	25	76.4 (7.3)	224	9.0 (3.3)
3a/45-60	57	74.3 (8.5)	365	6.4 (3.5)
1-2/>60	111	70.3 (10.2)	599	5.4 (2.9)
Total	198		1234	

S1 File shows the most commonly used drugs for patients with eGFR greater or less than 60.

The 87 patients with GFR less than 60 ml/min/1.73 m^2^ used a total of 635 medications. They detect 50 incidents (Medications that required review). This accounted for 7.9% of these medications (95% CI 5.8–10.0). Dose adjustment was requested in 31 and withdrawal in 19. Of these, 6 were adjusted (19.4% CI95% 5.5–33.3) and 8 were withdrawn (42.1% CI95% 19.9–64.3). The 14 medications that underwent modifications corresponded to 2.3% (95% CI; 1.1–3.4) of the medications used by patients with eGFR<60 ml/min/1.73 m ^2^, and 1.2% (95% CI; 0. 5–1.7) of the total drugs in the sample.

Table 4 lists all medicines that were requested for dose adjustment or withdrawal, and what was eventually done.

**Table 4:** Medicines for which a change is requested and the outcome of the request.

Adjustment requested				Withdrawal requested		
Active ingredient	n	Adjusted	Active ingredient		n	withdraws
METFORMINA	7	2	METFORMINA		1	1
SITAGLIPTINA	3	1	EMPAGLIFLOZINA		2	
VILDAGLIPTINA	1		COLECALCIFEROL		1	
ACETILSALICILICO, ACIDO (CARDIOLOGIA)	2		ACETILSALICILICO, ACIDO (CARDIOLOGIA)		1	
RANOLAZINA	1		HIDRALAZINA		1	1
HIDROCLOROTIAZIDA	3		HIDROCLOROTIAZIDA		2	
NEBIVOLOL	1		AMILORIDA		1	1
RAMIPRIL	1		BISOPROLOL		1	1
OLMESARTAN MEDOXOMILO	5		FENOFIBRATO		1	
ATORVASTATINA	2		POTASIO, CITRATO		1	
ALOPURINOL	1		DEXKETOPROFENO		1	1
PARACETAMOL	1		CELECOXIB		1	1
LEVETIRACETAM	1	1	ETORICOXIB		3	2
ZONISAMIDA	1	1	DULOXETINA		1	
KETAZOLAM	1	1	RUPATADINA		1	
Total	31	6	Total		19	8

The detail broken down by CKD stage can be seen in Table 5.

**Table 5:** Proposed changes and results obtained.

	Medicines					
	Proposed change			Change made		
CKD/ GF stage	Adjustment	Withdrawal	Total * No. (%)	Medication adjusted as suggested	Interrupted medication	Total** No. (%)
4–5 / <30	4	7	11 (23.9)	0	0	0 (0.0)
3b / 30–45	14	5	19 (8.5)	3	3	6 (31.6)
3a / 45–60	13	7	20 (5.5)	3	5	8 (40.0)
1–2 / >60	0	0	0	0	0	0
Total	31	19	50 (4.1)	6	8	14 (28.0)

* The percentage refers to the total number of drugs used by patients in the corresponding CKD group.

** The percentage refers to the number of proposed changes.

Table 6 lists the 6 medications with dose adjustment and the patient’s characteristics.

**Table 6:** Detail of the medications with dose adjustment and the patients who used them.

	Patient’s characteristics		
Drug	Sex	Age	eGFR
Metformin	Male	60	50
Sitagliptin			
Levetiracetam	Female	86	46
Ketazolam	Female	65	43
Zonisamide			
Metformin	Male	67	31

eGFR = Glomerular Filtration.

Table 7 lists the 8 medications that were discontinued and the patient’s characteristics.

**Table 7:** Detail of the medications that were discontinued and the patients who used them.

	Patient characteristics		
Drug	Sex	Age	eGFR
Etoricoxib	Female	90	59
Dexketoprofen	Male	76	57
Celecoxib	Male	89	46
Bisoprolol	Male	71	45
Metformin			
Etoricoxib	Female	78	44
Hydralazine	Female	65	43
Amiloride			

eGFR = Glomerular Filtration.

These 50 medications were taken by 29 patients, which represents 33.3% (95% CI 23.4–43.2) of the 87 patients with GFR<60. Nine patients had a change of treatment, representing 10.3% (95% CI 3.4–16.7) of the patients with GFR<60 and 4.6% (95% CI 1.6–7.5) of the total sample.

Tables 8–10 show the number of patients for whom a change is proposed and the details of the proposed change. It is important to note that the same patient may be proposed an adjustment of a medicine and a withdrawal of another.

**Table 8:** Number of patients for whom a change is proposed and those for whom a change is made.

	Patients	
CKD stage / eGFR (ml/min/1,73m2)	All changes proposed	Changes made
4–5 / <30	5	0
3b / 30–45	10	3
3a / 45–60	14	6
1–2 / >60	0	0
Total	29	9

**Table 9:** Number of patients for whom dose adjustment is proposed and those for whom it is adjusted.

	Proposed change	Change made
CKD stage / eGFR (ml/min/1,73m2)	Adjustment	Adjusted
4–5 / <30	4	0
3b / 30–45	10	2
3a / 45–60	9	2
1–2 / >60	0	0
Total	23	4

**Table 10:** Number of patients proposed for and withdrawn from any medicine.

	Proposed change	Change made
CKD stage / eGFR (ml/min/1,73m2)	Discontinuation	Discontinued
4–5 / <30	4	0
3b / 30–45	4	2
3a / 45–60	6	4
1–2 / >60	0	0
Total	14	6

Time spent caring for patients

Table 11 shows the time, in minutes, estimated by each pharmacy in caring for their patients.

**Table 11:** Time, in minutes, dedicated by each pharmacy to patient care.

	Pharmacy				Average time per patient	Number of patients	Total time
	1	2	3	4			
Patient not studied (eGFR > 60)	5	10	5	5	6.25	111	694
Patient studied (eGFR<60)	30	60	25	45	40	58	2320
Referred patient	45	80	40	55	55	29	1595
					Minutes		4609
					minutes / patient		23.28

The initial time dedicated to each patient was 6.25 minutes, which includes offering the service, signing the consent and carrying out the analysis. If a medication study was required, this time was extended by 33.75 minutes more. The patients who were referred for changes required an additional 15minutes. This includes writing the referral report and explaining it to the patient.

The net cost of one hour of work for a pharmacist, according to the current Labor Agreement, is €21.29. The average cost of work per patient is €8.26. To this must be added the cost of the test strip (€5.45) and the amortization of the device (estimated at €0.5 per analysis), which represents a total cost of €14.21 per patient. Broken down by patient, this represents a cost of €8.17 for non-studied patients, €20.14 for studied patients and €25.50 for referred patients.

There were 72 business days in the study period. This represents an average recruitment of 0.7 patients per pharmacy per day.

## Discussion

87 patients, 43.9% of the total, had an eGFR below 60 ml/min/1.73 m^2^ and therefore, given the limitation of having only a single measurement, were considered to have CKD. Our intervention cannot be considered a general screening because we have not considered a possible prior CKD diagnosis; our objective was focused on the patient’s medication. But positivity rate of 43.9% is much higher than generally reported in screening studies. Papastergiou J et al [28] found eGFR<60 of 11.1% in a study conducted in community pharmacies in Canada almost identical to that found by Donovan J et al 11% in a community pharmacy in Canada) [29]. These two studies included patients over 18 years old, with a mean age is 60 and 67 years, respectively. In Spain, Gorostidi M et al found eGFR<60 of 15.1% in the general population and 37.3% in people over 65 [4] In another study similar to ours carried out in 22 Spanish pharmacies [23] and patients older than 65 years, the incidence was 46.5% with eGFR<60 ml/min/1.73 m ^2^.

Such large differences are probably due to different inclusion criteria. In our case, limiting age has led to selecting older patients and, therefore, with a higher risk of CKD. This likely result in greater efficiency in both time and expenses.

As expected, patients with an eGFR<60 ml/min/1.73 m^2^ are older and take more medications. The latter factor, the number of drugs used, could be used as an inclusion criterion for further studies to seek greater efficiency, although this may also miss some patients with CKD.

Pharmacists reviewed all the medications taken by patients with eGFR <60ml/min/1.73m2 and found that 50(7.9%), posed a potential risk to the patient. This corresponded to 29 patients (33.3%). There are many studies that examine the adequacy of the prescribed dose based on the patient’s eGFR, but almost all of them are carried out in other settings, generally hospitals. We have only found one comparable to ours, the aforementioned Via Sosa [24]. In this study, carried out in two phases, they found that there were 9.6% and 8.7% of medications with dosing changes in each phase, values similar to ours.

These data are lower than those of a study [14] which reviewed medications in a cohort of 3033 patients with an eGFR <60 ml/min/1.73 m^2^ where it was found that 52% had received a contraindicated medication or an inappropriate dose. It accounts for 11.7% of the medicines prescribed to them. Here the prevalence is higher, perhaps because patients already diagnosed with CKD were studied.

Of the 29 patients in our study, the primary care doctor made changes in 9, representing 31.0% of the patients who underwent intervention, 10.3% of the patients with eGFR<60 and 4.5% of the total.

Interestingly, none of the changes proposed for patients with the most advanced stages of CKD, when eGFR<30 ml/min/1.73 m^2^, were implemented. Our interpretation of this is that these patients are more closely followed by their nephrologist and, therefore, although these drugs may pose a risk, the nephrologist considers that the benefit/risk balance is positive. There may also be other factors that were not evaluated in this study.

If we focus only on the patients with stage 3 CKD (eGFR between 30 and 60), we see that our intervention is more efficient as we achieve better results. The incidences are 39 medications, which represents 6.6% of the 589 medications used by these patients, but 14 of them are accepted, that is, 35.9% of the 39 medicines mentioned. This means that 24 patients in stage 3 have intervention and changes have been made in 9, 37.5% of the patients who underwent intervention and 11.0% of the 82 patients in stage 3.

Our work seems to be most effective in stage 3 CKD patients, but this does not mean that we should stop studying patients with stage 4 or 5 CKD because, although they are followed more closely, they are also more fragile.

To study the capacity of the pharmacy to develop this service, the time spent on each type of patient was estimated. To our knowledge no other study has evaluated this aspect. Our study showed that an average of 23 minutes was spent on each patient requiring testing. Given that these pharmacies were participating in a study for the first time, we consider it probable that an experienced pharmacist would need less time to carry it out.

On average, less than 1 patient recruited per day is a workload that should be easily assumed by most pharmacies. An average Spanish pharmacy employs 2.5 pharmacists per pharmacy [30]. Furthermore, if this service is implemented broadly, pharmacies will have a database of the *kidney* status of their regular patients, it would only be necessary to analyze the changes in treatment of patients with low eGFR. We´ve estimated that 23 minutes is the time spent by an inexperienced pharmacist reviewing a patient’s entire medication. We propose that an experienced pharmacist who only has to review changes in their patient’s treatment will spend much less time, probably half or less.

The results of this study are interesting, but 198 patients and 4 pharmacies are not enough to draw final conclusions. We consider that the feasibility of the service is proven and we showed that it can be done. But it is necessary to repeat the study with more patients and more pharmacies in order to specify more details. Establishing more precise selection criteria, agreed with primary care physicians, based on patient characteristics or treatment would make the work more efficient. Establishing a two-way communication channel between community pharmacy and primary care is critical. It is also important to agree on criteria for referral from pharmacy to primary care and, for this to be viable, some type of reimbursement for the pharmacy must be established.

The long-term objective and final goal of these studies is to demonstrate that the Spanish community pharmacy can efficiently collaborate with the Public Health System to benefit the health of the patients and decongest, to some extent, the public healthcare. Ideally, it would be very useful to be able to put these data in the patient’s clinical history so that any professional can have access to them, always with the patient’s authorization.

### Study limitations

The sample selection was not random. We selected people over 60 years of age who were prescribed a medication that could be adjusted or withdrawn. Due to its frequency and the risk inherent to its pathology, diabetic and/or hypertensive patients are overrepresented.

## Conclusions

During our study we have detected that 43.9% of patients had eGFR < 60ml/min/1.73m^2^. The implementation of Point-of-Care creatinine analyzer in community pharmacies was a critical element in identifying these patients.

We have concluded that, of the medications these patients were taking, 4.9% could require dose adjustment and 3.0% of the medications were nephrotoxic. In all those cases, dose adjustment and/or withdrawal were requested from the GP accordingly. The GPs adjusted 19.4% and withdrew 42.1% of the medications respectively. This represents 2.3% of all the drugs included in this study.

Globally, in our study, changes were proposed for the total of 14.6% of the patients and have been made in 31.0% of them. This represents 4.5% of the total number of patients included in the study.

We’ve also estimated the average time spent with each patient to be about 23 minutes and represents a cost of 14.21 euros per patient.

Importantly, we consider it necessary to establish a communication channel between the community pharmacy and the Primary Care system. We also consider necessary to reach a consensus with Primary Care professionals on the criteria for referral from the community pharmacy to Primary Care.

## Supporting information

S1 File (DOCX)

S2 File (DOCX)

S3 File (DOCX)

S4 File (DOCX)
