# Supplementary material for: Pharmaceutical care program for patients with chronic kidney disease in the community pharmacy: Detection of nephrotoxic drugs and dose adjustment. Viability study
Source: PLoS One. 2022 Dec 22;17(12):e0278648. doi: 10.1371/journal.pone.0278648 (PMC9778591; doi:10.1371/journal.pone.0278648)
Supplement: S1 File — (DOCX) [file pone.0278648.s001.docx]

Anex Medicines used by patients according to their eGFR ordered by frequency

| FG ≥ 60 mg/min/1,73m2 | | | | FG < 60 mg/min/1,73m2 | | | |
| --- | --- | --- | --- | --- | --- | --- | --- |
| Active ingredient | n | % | Cumulative % | Active ingredient | n | % | Cumulative % |
| OMEPRAZOL | 31 | 5,2% | 5,2% | ATORVASTATINA | 32 | 5,0% | 5,0% |
| ATORVASTATINA | 26 | 4,3% | 9,5% | OMEPRAZOL | 31 | 4,9% | 9,9% |
| PARACETAMOL | 26 | 4,3% | 13,9% | PARACETAMOL | 29 | 4,6% | 14,5% |
| METFORMINA | 21 | 3,5% | 17,4% | HIDROCLOROTIAZIDA | 28 | 4,4% | 18,9% |
| HIDROCLOROTIAZIDA | 20 | 3,3% | 20,7% | ACETILSALICILICO, ACIDO (CARDIOLOGIA) | 23 | 3,6% | 22,5% |
| ACETILSALICILICO, ACIDO (CARDIOLOGIA) | 20 | 3,3% | 24,0% | METFORMINA | 19 | 3,0% | 25,5% |
| LORAZEPAM | 16 | 2,7% | 26,7% | AMLODIPINO | 14 | 2,2% | 27,7% |
| BISOPROLOL | 14 | 2,3% | 29,0% | TAMSULOSINA | 13 | 2,0% | 29,8% |
| ENALAPRIL | 12 | 2,0% | 31,1% | OLMESARTAN MEDOXOMILO | 13 | 2,0% | 31,8% |
| VALSARTAN | 11 | 1,8% | 32,9% | TRAMADOL | 13 | 2,0% | 33,9% |
| SIMVASTATINA | 11 | 1,8% | 34,7% | SIMVASTATINA | 12 | 1,9% | 35,7% |
| LEVOTIROXINA | 11 | 1,8% | 36,6% | ALOPURINOL | 12 | 1,9% | 37,6% |
| TAMSULOSINA | 11 | 1,8% | 38,4% | BISOPROLOL | 11 | 1,7% | 39,4% |
| AMLODIPINO | 10 | 1,7% | 40,1% | ACENOCUMAROL | 10 | 1,6% | 40,9% |
| COLECALCIFEROL | 10 | 1,7% | 41,7% | LEVOTIROXINA | 10 | 1,6% | 42,5% |
| FUROSEMIDA | 9 | 1,5% | 43,2% | LORAZEPAM | 9 | 1,4% | 43,9% |
| OLMESARTAN MEDOXOMILO | 9 | 1,5% | 44,7% | VALSARTAN | 9 | 1,4% | 45,4% |
| DUTASTERIDA | 9 | 1,5% | 46,2% | FUROSEMIDA | 8 | 1,3% | 46,6% |
| CALCIO, CARBONATO | 7 | 1,2% | 47,4% | ROSUVASTATINA | 8 | 1,3% | 47,9% |
| DIAZEPAM | 7 | 1,2% | 48,6% | METAMIZOL | 8 | 1,3% | 49,1% |
| METAMIZOL | 7 | 1,2% | 49,7% | ESCITALOPRAM | 8 | 1,3% | 50,4% |
| TRAMADOL | 7 | 1,2% | 50,9% | FENOFIBRATO | 8 | 1,3% | 51,7% |
| IRBESARTAN | 7 | 1,2% | 52,1% | SITAGLIPTINA | 8 | 1,3% | 52,9% |
| PREGABALINA | 6 | 1,0% | 53,1% | COLECALCIFEROL | 8 | 1,3% | 54,2% |
| DEXKETOPROFENO | 6 | 1,0% | 54,1% | LINAGLIPTINA | 6 | 0,9% | 55,1% |
| TRAZODONA | 6 | 1,0% | 55,1% | ALPRAZOLAM | 6 | 0,9% | 56,1% |
| ACENOCUMAROL | 6 | 1,0% | 56,1% | FERROGLICINA,SULFATO | 6 | 0,9% | 57,0% |
| CALCIFEDIOL | 6 | 1,0% | 57,1% | RAMIPRIL | 6 | 0,9% | 58,0% |
| VILDAGLIPTINA | 6 | 1,0% | 58,1% | CALCIO, CARBONATO | 6 | 0,9% | 58,9% |
| LORMETAZEPAM | 5 | 0,8% | 58,9% | CANDESARTAN | 6 | 0,9% | 59,8% |
| TELMISARTAN | 5 | 0,8% | 59,8% | IRBESARTAN | 6 | 0,9% | 60,8% |
| ROSUVASTATINA | 5 | 0,8% | 60,6% | INSULINA GLARGINA | 6 | 0,9% | 61,7% |
| RAMIPRIL | 5 | 0,8% | 61,4% | PANTOPRAZOL | 6 | 0,9% | 62,7% |
| LOSARTAN | 5 | 0,8% | 62,3% | LORMETAZEPAM | 5 | 0,8% | 63,5% |
| EZETIMIBA | 4 | 0,7% | 62,9% | LANSOPRAZOL | 5 | 0,8% | 64,3% |
| ESCITALOPRAM | 4 | 0,7% | 63,6% | DILTIAZEM | 5 | 0,8% | 65,0% |
| PAROXETINA | 4 | 0,7% | 64,3% | ATENOLOL | 5 | 0,8% | 65,8% |
| IBUPROFENO | 4 | 0,7% | 64,9% | SOLIFENACINA | 5 | 0,8% | 66,6% |
| CLORAZEPATO DIPOTASICO | 4 | 0,7% | 65,6% | EZETIMIBA | 5 | 0,8% | 67,4% |
| CLOPIDOGREL | 4 | 0,7% | 66,3% | ETORICOXIB | 4 | 0,6% | 68,0% |
| RANITIDINA | 4 | 0,7% | 66,9% | ENALAPRIL | 4 | 0,6% | 68,7% |
| LERCANIDIPINO | 4 | 0,7% | 67,6% | NITROGLICERINA | 4 | 0,6% | 69,3% |
| PITAVASTATINA | 4 | 0,7% | 68,3% | MIRTAZAPINA | 4 | 0,6% | 69,9% |
| SERTRALINA | 4 | 0,7% | 68,9% | EMPAGLIFLOZINA | 4 | 0,6% | 70,6% |
| IMIDAPRIL | 3 | 0,5% | 69,4% | DIAZEPAM | 4 | 0,6% | 71,2% |
| FENOFIBRATO | 3 | 0,5% | 69,9% | DULOXETINA | 4 | 0,6% | 71,8% |
| FORMOTEROL | 3 | 0,5% | 70,5% | PENTOXIFILINA | 4 | 0,6% | 72,4% |
| FOLICO,ACIDO | 3 | 0,5% | 71,0% | BETAHISTINA | 4 | 0,6% | 73,1% |
| INDAPAMIDA | 3 | 0,5% | 71,5% | FLECAINIDA | 3 | 0,5% | 73,5% |
| ESOMEPRAZOL | 3 | 0,5% | 72,0% | RANOLAZINA | 3 | 0,5% | 74,0% |
| LANSOPRAZOL | 3 | 0,5% | 72,5% | LERCANIDIPINO | 3 | 0,5% | 74,5% |
| SITAGLIPTINA | 3 | 0,5% | 73,0% | REPAGLINIDA | 3 | 0,5% | 75,0% |
| ALENDRONICO, ACIDO | 3 | 0,5% | 73,5% | KETAZOLAM | 3 | 0,5% | 75,4% |
| TORASEMIDA | 3 | 0,5% | 74,0% | APIXABAN | 3 | 0,5% | 75,9% |
| TIOTROPIO, BROMURO | 3 | 0,5% | 74,5% | RABEPRAZOL | 3 | 0,5% | 76,4% |
| AMOXICILINA | 3 | 0,5% | 75,0% | CONDROITIN SULFATO (CARDIOVASCULAR) | 3 | 0,5% | 76,9% |
| ATENOLOL | 3 | 0,5% | 75,5% | CETIRIZINA | 3 | 0,5% | 77,3% |
| EMPAGLIFLOZINA | 3 | 0,5% | 76,0% | AMIODARONA | 3 | 0,5% | 77,8% |
| CANDESARTAN | 3 | 0,5% | 76,5% | DUTASTERIDA | 3 | 0,5% | 78,3% |
| DULOXETINA | 3 | 0,5% | 77,0% | TORASEMIDA | 3 | 0,5% | 78,7% |
| DABIGATRAN ETEXILATO | 3 | 0,5% | 77,5% | ESOMEPRAZOL | 3 | 0,5% | 79,2% |
| OLODATEROL | 3 | 0,5% | 78,0% | ZOLPIDEM | 3 | 0,5% | 79,7% |
| REPAGLINIDA | 3 | 0,5% | 78,5% | CIANOCOBALAMINA | 3 | 0,5% | 80,2% |
| DESVENLAFAXINA | 2 | 0,3% | 78,8% | PRAVASTATINA | 3 | 0,5% | 80,6% |
| DIGOXINA | 2 | 0,3% | 79,1% | PREGABALINA | 3 | 0,5% | 81,1% |
| ALOPURINOL | 2 | 0,3% | 79,5% | EPROSARTAN | 3 | 0,5% | 81,6% |
| ZOLPIDEM | 2 | 0,3% | 79,8% | PIOGLITAZONA | 3 | 0,5% | 82,0% |
| BETAHISTINA | 2 | 0,3% | 80,1% | BUDESONIDA (INHALADO) | 2 | 0,3% | 82,4% |
| ALPRAZOLAM | 2 | 0,3% | 80,5% | DEXKETOPROFENO | 2 | 0,3% | 82,7% |
| APIXABAN | 2 | 0,3% | 80,8% | SALBUTAMOL | 2 | 0,3% | 83,0% |
| CONDROITIN SULFATO (CARDIOVASCULAR) | 2 | 0,3% | 81,1% | FOLICO,ACIDO | 2 | 0,3% | 83,3% |
| INSULINA ASPART | 2 | 0,3% | 81,5% | DIOSMINA | 2 | 0,3% | 83,6% |
| CINITAPRIDA | 2 | 0,3% | 81,8% | PITAVASTATINA | 2 | 0,3% | 83,9% |
| CARBOCISTEINA | 2 | 0,3% | 82,1% | DOXAZOSINA | 2 | 0,3% | 84,3% |
| GLUCOSAMINA | 2 | 0,3% | 82,5% | AMILORIDA | 2 | 0,3% | 84,6% |
| PRAVASTATINA | 2 | 0,3% | 82,8% | NEBIVOLOL | 2 | 0,3% | 84,9% |
| POTASIO, CLORURO (ORAL) | 2 | 0,3% | 83,1% | SALICILICO,ACIDO | 2 | 0,3% | 85,2% |
| SALBUTAMOL | 2 | 0,3% | 83,5% | BROMAZEPAM | 2 | 0,3% | 85,5% |
| RABEPRAZOL | 2 | 0,3% | 83,8% | DAPAGLIFLOZINA | 2 | 0,3% | 85,8% |
| SOLIFENACINA | 2 | 0,3% | 84,1% | ANASTROZOL | 2 | 0,3% | 86,1% |
| IVABRADINA | 2 | 0,3% | 84,5% | ALENDRONICO, ACIDO | 2 | 0,3% | 86,5% |
| PANTOPRAZOL | 2 | 0,3% | 84,8% | TRAZODONA | 2 | 0,3% | 86,8% |
| RUPATADINA | 2 | 0,3% | 85,1% | VILDAGLIPTINA | 2 | 0,3% | 87,1% |
| PIRIDOXINA | 2 | 0,3% | 85,5% | CALCIFEDIOL | 2 | 0,3% | 87,4% |
| MIRABEGRON | 2 | 0,3% | 85,8% | PAROXETINA | 2 | 0,3% | 87,7% |
| RISPERIDONA | 2 | 0,3% | 86,1% | PREDNISONA | 2 | 0,3% | 88,0% |
| CALCIO,FOSFATO | 1 | 0,2% | 86,3% | BEMIPARINA | 1 | 0,2% | 88,2% |
| CEFDITORENO | 1 | 0,2% | 86,5% | ENOXAPARINA | 1 | 0,2% | 88,3% |
| CARBAMAZEPINA | 1 | 0,2% | 86,6% | BIOTINA | 1 | 0,2% | 88,5% |
| RIFAXIMINA | 1 | 0,2% | 86,8% | ALOGLIPTINA | 1 | 0,2% | 88,7% |
| CARVEDILOL | 1 | 0,2% | 87,0% | CLARITROMICINA | 1 | 0,2% | 88,8% |
| CEFUROXIMA | 1 | 0,2% | 87,1% | CALCIO, ACETATO | 1 | 0,2% | 89,0% |
| CICLOBENZAPRINA | 1 | 0,2% | 87,3% | CALCIO, CARBONATO (ANTIACIDO) | 1 | 0,2% | 89,1% |
| RASAGILINA | 1 | 0,2% | 87,5% | AZITROMICINA | 1 | 0,2% | 89,3% |
| CITALOPRAM | 1 | 0,2% | 87,6% | ASCORBICO, ACIDO | 1 | 0,2% | 89,4% |
| CLAVULANICO,ACIDO, POTASIO (SAL | 1 | 0,2% | 87,8% | BAZEDOXIFENO | 1 | 0,2% | 89,6% |
| CLORFENAMINA | 1 | 0,2% | 88,0% | CEFUROXIMA | 1 | 0,2% | 89,8% |
| CODEINA | 1 | 0,2% | 88,1% | DENOSUMAB | 1 | 0,2% | 89,9% |
| COLCHICINA | 1 | 0,2% | 88,3% | CAFEINA | 1 | 0,2% | 90,1% |
| PRAMIPEXOL | 1 | 0,2% | 88,5% | AMITRIPTILINA | 1 | 0,2% | 90,2% |
| CIANOCOBALAMINA | 1 | 0,2% | 88,6% | CLEBOPRIDA | 1 | 0,2% | 90,4% |
| ASCORBICO, ACIDO | 1 | 0,2% | 88,8% | CLORPROMAZINA | 1 | 0,2% | 90,6% |
| TRIMETOPRIM | 1 | 0,2% | 89,0% | CODEINA | 1 | 0,2% | 90,7% |
| ACETOHIDROXAMICO, ACIDO | 1 | 0,2% | 89,1% | AMOXICILINA | 1 | 0,2% | 90,9% |
| ADALIMUMAB | 1 | 0,2% | 89,3% | CONDROITIN SULFATO (OSTEOMUSCULAR) | 1 | 0,2% | 91,0% |
| TRIFLUSAL | 1 | 0,2% | 89,5% | CINITAPRIDA | 1 | 0,2% | 91,2% |
| TEOFILINA | 1 | 0,2% | 89,6% | CELECOXIB | 1 | 0,2% | 91,3% |
| TAPENTADOL | 1 | 0,2% | 89,8% | RIVASTIGMINA | 1 | 0,2% | 91,5% |
| SULPIRIDA | 1 | 0,2% | 90,0% | METOTREXATO (ANTINEOPLASICO) | 1 | 0,2% | 91,7% |
| SULFAMETOXAZOL | 1 | 0,2% | 90,2% | METRONIDAZOL (ORAL) | 1 | 0,2% | 91,8% |
| CALCIO, ACETATO | 1 | 0,2% | 90,3% | MIRABEGRON | 1 | 0,2% | 92,0% |
| ARIPIPRAZOL | 1 | 0,2% | 90,5% | MONTELUKAST | 1 | 0,2% | 92,1% |
| BUDESONIDA (INHALADO) | 1 | 0,2% | 90,7% | OXIBUTININA | 1 | 0,2% | 92,3% |
| SILDENAFILO (UROLOGIA) | 1 | 0,2% | 90,8% | PALIPERIDONA | 1 | 0,2% | 92,4% |
| BARNIDIPINO | 1 | 0,2% | 91,0% | PIRIDOXINA | 1 | 0,2% | 92,6% |
| BECLOMETASONA (ENEMA) | 1 | 0,2% | 91,2% | POTASIO,CITRATO | 1 | 0,2% | 92,8% |
| SAXAGLIPTINA | 1 | 0,2% | 91,3% | PRAMIPEXOL | 1 | 0,2% | 92,9% |
| BILASTINA | 1 | 0,2% | 91,5% | PREDNISOLONA | 1 | 0,2% | 93,1% |
| BIMATOPROST | 1 | 0,2% | 91,7% | RASAGILINA | 1 | 0,2% | 93,2% |
| SALMETEROL | 1 | 0,2% | 91,8% | HIDROSMINA | 1 | 0,2% | 93,4% |
| BROMAZEPAM | 1 | 0,2% | 92,0% | RIVAROXABAN | 1 | 0,2% | 93,5% |
| BUDESONIDA (A07EA) | 1 | 0,2% | 92,2% | LEVODOPA | 1 | 0,2% | 93,7% |
| SULBUTIAMINA | 1 | 0,2% | 92,3% | ROPINIROL | 1 | 0,2% | 93,9% |
| GLUCOSA | 1 | 0,2% | 92,5% | RUPATADINA | 1 | 0,2% | 94,0% |
| CONDROITIN SULFATO (OSTEOMUSCULAR) | 1 | 0,2% | 92,7% | SILODOSINA | 1 | 0,2% | 94,2% |
| FLUCONAZOL | 1 | 0,2% | 92,8% | SIMETICONA | 1 | 0,2% | 94,3% |
| FLUOXETINA | 1 | 0,2% | 93,0% | SULBUTIAMINA | 1 | 0,2% | 94,5% |
| FLUTICASONA (INHALADO) | 1 | 0,2% | 93,2% | TAPENTADOL | 1 | 0,2% | 94,6% |
| FLUVASTATINA | 1 | 0,2% | 93,3% | TELMISARTAN | 1 | 0,2% | 94,8% |
| MEMANTINA | 1 | 0,2% | 93,5% | TIAMINA | 1 | 0,2% | 95,0% |
| LISINOPRIL | 1 | 0,2% | 93,7% | TIOTROPIO, BROMURO | 1 | 0,2% | 95,1% |
| FOSFOMICINA | 1 | 0,2% | 93,8% | TOPIRAMATO | 1 | 0,2% | 95,3% |
| LEVETIRACETAM | 1 | 0,2% | 94,0% | TRIFLUSAL | 1 | 0,2% | 95,4% |
| FESOTERODINA | 1 | 0,2% | 94,2% | RISEDRONICO, ACIDO | 1 | 0,2% | 95,6% |
| GLICOPIRRONIO, BROMURO (INHALADO) | 1 | 0,2% | 94,3% | ACECLOFENACO | 1 | 0,2% | 95,7% |
| METOTREXATO (ANTINEOPLASICO) | 1 | 0,2% | 94,5% | EXENATIDA | 1 | 0,2% | 95,9% |
| LATANOPROST | 1 | 0,2% | 94,7% | FENTANILO (ANESTESICO) | 1 | 0,2% | 96,1% |
| LAMOTRIGINA | 1 | 0,2% | 94,8% | FENTANILO (TRANSDERMICO) | 1 | 0,2% | 96,2% |
| LACOSAMIDA | 1 | 0,2% | 95,0% | FESOTERODINA | 1 | 0,2% | 96,4% |
| KETAZOLAM | 1 | 0,2% | 95,2% | FINASTERIDA (ANTIADENOMA PROSTATICO) | 1 | 0,2% | 96,5% |
| INDACATEROL | 1 | 0,2% | 95,3% | FLUTICASONA, FUROATO (NASAL) | 1 | 0,2% | 96,7% |
| IPRATROPIO, BROMURO (NASAL) | 1 | 0,2% | 95,5% | FORMOTEROL | 1 | 0,2% | 96,9% |
| INSULINA LISPRO | 1 | 0,2% | 95,7% | FOSFOMICINA | 1 | 0,2% | 97,0% |
| INSULINA DEGLUDEC | 1 | 0,2% | 95,8% | FOSINOPRIL | 1 | 0,2% | 97,2% |
| INSULINA GLARGINA | 1 | 0,2% | 96,0% | GABAPENTINA | 1 | 0,2% | 97,3% |
| GLICLAZIDA | 1 | 0,2% | 96,2% | GLICLAZIDA | 1 | 0,2% | 97,5% |
| NEBIVOLOL | 1 | 0,2% | 96,3% | MEDAZEPAM | 1 | 0,2% | 97,6% |
| DAPAGLIFLOZINA | 1 | 0,2% | 96,5% | HIDRALAZINA | 1 | 0,2% | 97,8% |
| INSULINA GLULISINA | 1 | 0,2% | 96,7% | LISINOPRIL | 1 | 0,2% | 98,0% |
| DENOSUMAB | 1 | 0,2% | 96,8% | HIERRO (II), CARBONATO | 1 | 0,2% | 98,1% |
| PERINDOPRIL | 1 | 0,2% | 97,0% | ZONISAMIDA | 1 | 0,2% | 98,3% |
| DEXTROMETORFANO | 1 | 0,2% | 97,2% | HIERRO (II), SUCCINATO | 1 | 0,2% | 98,4% |
| OXICODONA | 1 | 0,2% | 97,3% | HIERRO (III)-AMONIO,CITRATO | 1 | 0,2% | 98,6% |
| OLANZAPINA | 1 | 0,2% | 97,5% | INDAPAMIDA | 1 | 0,2% | 98,7% |
| DILTIAZEM | 1 | 0,2% | 97,7% | INSULINA | 1 | 0,2% | 98,9% |
| DOXAZOSINA | 1 | 0,2% | 97,8% | INSULINA ASPART | 1 | 0,2% | 99,1% |
| FLECAINIDA | 1 | 0,2% | 98,0% | INSULINA GLULISINA | 1 | 0,2% | 99,2% |
| NITRENDIPINO | 1 | 0,2% | 98,2% | INSULINA ISOFANICA | 1 | 0,2% | 99,4% |
| HIERRO (II), CARBONATO | 1 | 0,2% | 98,3% | ISOSORBIDA, DINITRATO | 1 | 0,2% | 99,5% |
| EBASTINA | 1 | 0,2% | 98,5% | LEVETIRACETAM | 1 | 0,2% | 99,7% |
| NALOXONA | 1 | 0,2% | 98,7% | ESPIRONOLACTONA | 1 | 0,2% | 99,8% |
| MORFINA | 1 | 0,2% | 98,8% | GLIMEPIRIDA | 1 | 0,2% | 100,0% |
| EPLERENONA | 1 | 0,2% | 99,0% | Total | 635 |  |  |
| EPROSARTAN | 1 | 0,2% | 99,2% |  |  |  |  |
| MONTELUKAST | 1 | 0,2% | 99,3% |  |  |  |  |
| MIRTAZAPINA | 1 | 0,2% | 99,5% |  |  |  |  |
| ETORICOXIB | 1 | 0,2% | 99,7% |  |  |  |  |
| FENILEFRINA | 1 | 0,2% | 99,8% |  |  |  |  |
| DOXILAMINA | 1 | 0,2% | 100,0% |  |  |  |  |
| Total | 599 |  |  |  |  |  |  |
