# Supplementary material for: Pharmaceutical care program for patients with chronic kidney disease in the community pharmacy: Detection of nephrotoxic drugs and dose adjustment. Viability study
Source: PLoS One. 2022 Dec 22;17(12):e0278648. doi: 10.1371/journal.pone.0278648 (PMC9778591; doi:10.1371/journal.pone.0278648)
Supplement: S2 File — (DOCX) [file pone.0278648.s002.docx]

**INFORMED CONSENT FORM**

| **TítLE** | **Pharmaceutical care program for patients with chronic kidney disease in the community pharmacy: Detection of nephrotoxic drugs and dose adjustment. Viability study.** |
| --- | --- |

I, *……………………………………………………………………………………………………*

⎕ I have read the information sheet I have been given about the study..

⎕ I was able to ask questions about the study

⎕ I have received sufficient information about the study.

⎕ I have spoken to <<name of researcher>>

⎕ I understand that my participation is voluntary.

⎕ I understand that I can withdraw from the study:

- Whenever I want.

- Without having to explain myself.

- Without affecting my medical care.

.

| I will receive a signed and dated copy of this informed consent document.  I freely agree to participate in the study.. |  |
| --- | --- |
| \| \| Participant's signature \| Researcher’s signature \| \| --- \| --- \| \| Date: ____/____/____ \| Date: ____/____/____ \| \| \| \| \| --- \| --- \| --- \| --- \| --- \| --- \| --- \| \| (Name, signature and date in the patient's own handwriting)  * When the ICF is obtained in persons with modified capacity to give their ICF.   \| Legal representant signature Researcher’s signature  Date: ____/____/____ Date: ____/____/____ \| \| --- \| \| \|  \| \|  \| \|  \| | |
