# Supplementary material for: Pharmaceutical care program for patients with chronic kidney disease in the community pharmacy: Detection of nephrotoxic drugs and dose adjustment. Viability study
Source: PLoS One. 2022 Dec 22;17(12):e0278648. doi: 10.1371/journal.pone.0278648 (PMC9778591; doi:10.1371/journal.pone.0278648)
Supplement: S3 File — (DOCX) [file pone.0278648.s003.docx]

**PATIENT INFORMATION SHEET**

| **Títle** | **Pharmaceutical care program for patients with chronic kidney disease in the community pharmacy: Detection of nephrotoxic drugs and dose adjustment. Viability study.** |
| --- | --- |
| **Sponsor** | Sociedad Española de Farmacia Familiar y Comunitaria. Delegación de la Comunidad Valenciana.  Paseo de las Delicias, 31 - Esc. Izd. 4º Dcha28045 Madrid |
| Principal Investigator | Verónica Escudero Quesada  Gema Escriba Martí |
| **Institution** | **HOSPITAL UNIVERSITARI DR. PESET DE VALÈNCIA** |
| **Contact telephone number** | **+34687938779** |

**INTRODUCTION**

We are writing to inform you about a research study in which you are invited to participate. The study has been approved by the Ethics Committee for Research with Medicines (CEIm) of the Hospital Universitari Dr. Peset de València, in accordance with current legislation and the standards of good clinical practice.

Our intention is that you receive correct and sufficient information so that you can decide whether or not to participate in this study. To this end, please read this information sheet carefully and we will clarify any doubts you may have. You can also consult with the people you consider appropriate.

Chronic kidney disease (CKD) is common in our environment.

In these patients, the use of medication can lead to risky situations. There are drugs that can cause acute kidney damage after use, these are nephrotoxic drugs.

In addition, there are other drugs in which it is important to review the dose according to the renal status of each patient, because if the dose is not the right dose and it is the toxic dose, it can lead to an unsafe situation and adverse effects of the drug.

Both are risk situations for patients with CKD that can affect renal function and worsen the disease.

**VOLUNTARY PARTICIPATION**

You should be aware that your participation in this study is voluntary and that you may decide NOT to participate. If you decide to participate, you may change your decision and withdraw your consent at any time, without altering your relationship with your doctor or harming your health care.

**AIM OF THE STUDY**

To evaluate the effectiveness of the community pharmacist in the detection of nephrotoxic drugs and in the resolution of inappropriate drug dosage, after the implementation of a pharmaceutical care programme for patients with chronic kidney disease in community pharmacies, in order to improve the level of such care.

**DESCRIPTION OF THE STUDY**

This study aims to improve the level of care in community pharmacies for patients with chronic kidney disease. The pharmacist will assess the patient's medications to detect these two risk situations, nephrotoxic drugs and inadequate doses of drugs, and in this case inform the doctor.

To assess the medicines, the pharmacist will conduct a brief interview with the patient in a personalised care area. The purpose of the interview is to obtain information about the medicines taken and how they are taken in order to detect these two risk situations.

Several community pharmacies will assess the target population who come to the participating community pharmacies to pick up their medication. The population included will be over 60 years of age, with estimated glomerular filtration rate < 60 ml/min, using at least one of the following drugs: antidiabetic, antihypertensive, lipid-lowering, involved in the treatment of obstructive diseases of the urinary tract, nephrotoxic. Number of subjects to be included: 200. The aim is to evaluate the effectiveness of the pharmacist in the detection of inadequate drug dosage and detection of nephrotoxic drugs in patients with chronic kidney disease.

**STUDY ACTIVITIES**

During 3 months, data will be collected from the population visiting the participating community pharmacies, who meet the inclusion criteria (determining the glomerular filtration rate if necessary by capillary blood analysis in the pharmacy): review of potential drugs likely to be nephrotoxic or to adjust their dose to the patient's glomerular filtration rate, and prepare a report for their primary care physician.

RISKS AND DISCOMFORTS OF PARTICIPATION

The only inconvenience of the study may be the taking of a capillary blood sample from the finger to determine renal function if not previously known.

**POSSIBLE BENEFITS**

Uncovering possible previously unknown occult kidney disease, with the impact on cardiovascular risk and drug adjustment that may result.

**ALTERNATIVE TREATMENTS**

Not applicable.

**INSURANCE**

Not required.

**CONFIDENTIALITY**

The processing, communication and transfer of the personal data of all participating subjects will comply with the provisions of Organic Law 3/2018, of 5 December, on the Protection of Personal Data and the guarantee of digital rights.

In accordance with the provisions of data protection legislation, you may exercise your rights of access, modification, opposition and cancellation of data by contacting your study doctor.

In addition to the above rights, and in application of Regulation (EU) 2016/679 of the European Parliament and of the Council of 27 April 2016 on Data Protection, you can now also limit the processing of data that is incorrect by requesting a copy or that the data you have provided for the study be transferred to a third party (portability).

If you stop participating in the study, the data collected cannot be deleted in order to ensure the validity of the research. You also have the right to contact the Data Protection Agency if you are not satisfied.

Both the Centre and the Promoter are respectively responsible for the processing of your data and undertake to comply with the data protection regulations in force. The data collected for the study will be identified by a code, so that no information that can identify you is included, and only your study doctor/collaborators will be able to relate this data to you and your medical history. Therefore, your identity will not be disclosed to any other person except to health authorities, when required or in cases of medical emergency. Research Ethics Committees, representatives of the inspecting Health Authority and personnel authorised by the Sponsor may only have access to verify personal data, clinical trial procedures and compliance with the standards of good clinical practice (while maintaining the confidentiality of the information).

The Investigator and the Sponsor are obliged to retain the data collected for the study for at least 25 years after completion of the study. Thereafter, your personal information will only be retained by the Facility for your health care and by the Sponsor for other scientific research purposes if you have given your consent to do so, and if permitted by applicable law and ethical requirements.

If we transfer your encrypted data outside the European Union, the participant's data will be protected with safeguards such as contracts or other mechanisms by data protection authorities.

**FINANCIAL COMPENSATION**

"No financial compensation of any kind is envisaged for the subjects participating in the study, nor for the research team and the centre".

**OTHER RELEVANT INFORMATION**

By signing the attached consent form, you agree to comply with the study procedures outlined to you.

**CONTACT DETAILS**

If during your participation you have any doubts or need further information, please contact Gema Escribá Martí. Calle Mayor, 8 C.P.46592 Segart Valencia
