# Supplementary material for: Pharmaceutical care program for patients with chronic kidney disease in the community pharmacy: Detection of nephrotoxic drugs and dose adjustment. Viability study
Source: PLoS One. 2022 Dec 22;17(12):e0278648. doi: 10.1371/journal.pone.0278648 (PMC9778591; doi:10.1371/journal.pone.0278648)
Supplement: S4 File — (DOCX) [file pone.0278648.s004.docx]

**PHARMACOTHERAPEUTIC REPORT FOR REFERRAL TO THE PRIMARY CARE PHYSICIAN**

Pharmacy:……………………………………………………………………………..……Date:……/……/……

Adress:………………………………………………………………………………………………………..……..

Phone number:……………………………e-mail:………………………………..web:………………………….

Dear Dr.

Following the participation of Mr./Mrs./.............................................................................................., aged.......... years, in the research project entitled *Pharmaceutical care program for patients with chronic kidney disease in the community pharmacy: Detection of nephrotoxic drugs and dose adjustment. Viability study.*, which aims to improve the level of care for these patients, in the use of their medications according to their renal status, I have determined the creatinine with dry chemistry, the result has been........... mg/dl (1) which corresponds to an estimated glomerular filtration value of........... ml/min/1.73 m2 (2).

If this glomerular filtration rate is confirmed, the following drugs would need a dose adjustment to match his renal function

| Drug | Prescribed daily dose | Proposed daily dose |
| --- | --- | --- |
|  |  |  |
|  |  |  |
|  |  |  |
|  |  |  |
|  |  |  |
|  |  |  |
|  |  |  |

In addition, the following drugs are nephrotoxic and may cause acute kidney injury

| 1 |  |
| --- | --- |
| 2 |  |
| 3 |  |
| 4 |  |
| 5 |  |
| 6 |  |

I am therefore sending it to you so that you can assess the patient and proceed as you deem appropriate.

Please inform me of your decision so that I can record it correctly in the above-mentioned study.

Yours sincerely

(Name, surname and membership number)

^(1) Creatinine value mg/dl determined with StatSensorXpress®;^

^(2) Estimated glomerular filtration rate ml/min/1.73 m2 using the CKD-EPI equation;^
